# 

**Published:** 1902-12

**Authors:** 


### The Library of the Bristol Medico-Chirurgical Society.

*The following donations have been received since the publication of the List in September:*

*November 30th, 1902.*

| | |
|---|---|
| The Boston Medical Library (1) ... ... ... ... | 82 volumes. |
| A. L. Flemming (2) ... ... ... ... ... ... ... ... ... | 29 ,, |
| L. M. Griffiths (3) ... ... ... ... ... ... ... ... ... | 10 ,, |
| The Medical Library, McGill University (4) ... | 1 volume. |
| The Medico-Psychological Association of Great Britain and Ireland (5) ... ... ... ... ... ... | 1 ,, |
| Dr. B. M. H. Rogers (6) ... ... ... ... ... ... ... | 2 volumes. |
| The Editor of the "Virginia Medical Semi-Monthly" (7) ... ... ... ... ... ... ... ... | 1 volume. |

An unframed portrait has been presented by the Family of the late Dr. Neale, and unbound periodicals have been received from the Boston Medical Library, Mr. L. M. Griffiths, and Dr. B. M. H. Rogers.

### FORTY-SIXTH LIST OF BOOKS.

The titles of books mentioned in previous lists are not repeated.

The figures in brackets refer to the figures after the names of the donors, and show by whom the volumes were presented. The books to which no such figures are attached have either been bought from the Library Fund or received through the *Journal*.

**Allchin, W. H.** [Ed.] *A Manual of Medicine*... ... ... ... ... Vol. IV. 1902

**Beasley, H.**... ... *The Pocket Formulary* ... ... ... ... ... (2) 5th Ed. 1851

**Berry, R. J. A.** ... *Regional Anatomy.* 3 vols. ... ... New [2nd] Ed. 1902
**Björkstén, J. I.** ... *Vaccinationens Historia i Finland* ... ... ... ... ... ... 1902
**Bland-Sutton, J.** ... *See* **Sutton.**
**Blumfeld, J.** ... *Anæsthetics* ... ... ... ... ... ... ... ... ... ... 1902
**Carless and W. Rose, A.** *A Manual of Surgery* ... ... ... ... 5th Ed. 1902
**Carpenter, W. B.** *Human Physiology* ... ... ... ... ... ... (2) 3rd Ed. 1846
**Carr, T. P. Pick, A. H. G. Doran and A. Duncan, J. W.** *The Practitioner's Guide* ... ... ... ... ... ... ... ... ... ... 1902
*Catalogue of the Specimens illustrating Surgical Pathology in the Museum of University College, London,* Parts i., ii. ...(6) 1881–87
**Charcot, [J. M.]** ... *Clinique des Maladies du Système nerveux.* Tome ii. (3) 1893
**Churchill, F.** ... *Diseases of Women* ... ... ... ... ... ... (2) 3rd Ed. 1850
,, ... *Midwifery* ... ... ... ... ... ... ... (2) 2nd Ed. 1850
**Cole, J. J.** ... ... *Military Surgery* ... ... ... ... ... ... ... (2) 1852
**Coles, A. C.** ... ... *The Blood* ... ... ... ... ... ... ... ... 2nd Ed. 1902
**Corfield, W. H.** ... *Typhoid Fever* ... ... ... ... ... ... ... ... ... ... 1902
**Doran and A. Duncan, J. W. Carr, T. P. Pick, A. H. G.** *The Practitioner's Guide* ... ... ... ... ... ... ... ... ... ... 1902
**Druitt, R.** ... ... *The Surgeon's Vade-Mecum* (2) 2nd Ed., 1841, (2) 6th Ed. 1854
**Duncan, J. W. Carr, T. P. Pick, A. H. G. Doran and A.** *The Practitioner's Guide* ... ... ... ... ... ... ... ... ... ... 1902
**Ede, R. B.** ... ... *Practical Facts in Chemistry* ... ... ... ... ... ... (3) 1837
**Ekgren, E.** ... ... *Taschenbuch der Massage* ... ... ... ... 1903 [*sic*]
*Encyclopædia Medica.* (Ed. by C. Watson). Vol. XI. ... ... ... ... ... 1902
**Eyre, J. W. H.** ... *The Elements of Bacteriological Technique* ... ... ... 1902
**Fuller, H. W.** ... *On Rheumatism, Rheumatic Gout, and Sciatica* ... (2) 1852
**Gould and J. C. Warren, A. P.** [Eds.] *The International Text-Book of Surgery.* Vol. II. ... ... ... ... ... ... ... ... 1900
**Grant, H. H.** ... *A Text-Book of Surgical Principles and Surgical Diseases of the Face, Mouth, and Jaws* ... ... ... ... 1902
**Guibert, P.** ... ... *The Charitable Physitian, with the Charitable Apothecary* (Tr. by J. W.) ... ... ... ... ... ... ... (3) 1639
**Haig, A.** ... ... *Diet and Food* ... ... ... ... ... ... ... 4th Ed. 1902
**Hamer, W. H.** ... *Manual of Hygiene* ... ... ... ... ... ... ... ... 1902
**Heath, [C.]** ... ... *Practical Anatomy* (Ed. by J. E. Lane) ... 9th Ed. 1902
**Hemmeter, J. C.** ... *Diseases of the Intestines* ... ... ... ... 2 vols. 1901–02
*Holmes, Life and Letters of.* By J. T. Morse, Jr. 2 vols. ... ... ... 1896
**Hutchinson, J.** ... *An Atlas of Illustrations of Clinical Medicine, Surgery, and Pathology* ... ... ... ... ... ... ... ... 1902
**Kayser, R.** ... ... *Kehlkopf-, Nasen- und Ohrenkrankheiten.* 2nd Ed. 1903 [*sic*]
**Kenwood, L. Parkes and H.** *Hygiene and Public Health.* ... ... 2nd Ed. 1902
**Kirkby, W.** ... ... *The Evolution of Artificial Mineral Waters* ... ... ... 1902
**Knuthsen, L. F. B.** *Obstinate Hiccough* ... ... ... ... ... ... ... ... ... 1902
**Latham, P. M.** ... *Diseases of the Heart.* 2 vols. ... ... ... ... (3) 1846
**Laurence, J. L.** ... *Surgical Cancer* ... ... ... ... ... ... ... ... (2) 1855
**Leech, D. J.** ... ... *Nitrites and Allied Compounds.* (Ed. by R. B. Wild) 1902
**Lewers, A. H. N.** *Cancer of the Uterus* ... ... ... ... ... ... ... ... 1902
**Mattison, J. B.** ... *The Mattison Method in Morphinism* ... ... ... ... 1902

**Meade, [W.]** ... *Manual for Students* ... ... ... ... ... (2) 2nd Ed. 1846
**Moor, C. G.**... ... *Suggested Standards of Purity for Foods and Drugs* ... 1902
**Morse, J. T. (Jr.)** *Life and Letters of Oliver Wendell Holmes.* 2 vols. ... 1896
**Moynihan, A. W. M. Robson and B. G. A.** *Diseases of the Pancreas* ... 1902
**Muir and J. Ritchie, R.** *Manual of Bacteriology* ... ... ... ... 3rd Ed. 1902
**Natier, M.** ... ... *Voix de Faussett* ... ... ... .. ... ... ... ... 1902
**Oliver, T.** [Ed.] ... *Dangerous Trades* ... ... ... ... ... ... ... ... 1902
**Parkes and H. Kenwood, L.** *Hygiene and Public Health* ... ... 2nd Ed. 1902
**Phillips, R.**... ... *A Translation of the Pharmacopœia of the Royal College of Physicians of London*, 1836 .. (2) 4th Ed. 1841
**Pick, A. H. G. Doran and A. Duncan, J. W. Carr, T. P.** *The Practitioner's Guide* ... ... ... ... ... ... ... ... ... ... ... 1902
**Reid, J.** ... ... *Manual of Practical Midwifery* ... .. ... ... (2) 1836
**Ritchie, R. Muir and J.** *Manual of Bacteriology* ... ... ... .. 3rd Ed. 1902
**Robson and B. G. A. Moynihan, A. W. M.** *Diseases of the Pancreas* ... 1902
**Rose and A. Carless, W.** *A Manual of Surgery* ... ... ... ... 5th Ed. 1902
**Saundby, R.** ... *Medical Ethics*... ... ... .. ... ... ... ... ... 1902
**Smedley, J.** ... ... *Practical Hydropathy* ... ... ... ... ... ... (2) 1861
**Solari, B. T.** ... *Degeneración y Crímen* ... ... ... ... ... 2nd Ed. 1901
**Steele, W. E.** ... *Field Botany* ... ... ... ... ... ... ... ... (2) 1847
**Steggall, J.**... ... *Medical Manual* ... ... ... ... ... ... (2) 11th Ed. 1852
,, ... ... *Surgical Manual* ... ... .. ... ... (2) 2nd Ed. 1853
**Stelwagon, H. W.** *Treatise on Diseases of the Skin* .. ... ... ... ... 1902
**Stephenson, S.** ... *Ophthalmic Nursing* ... ... ... ... 2nd Ed. [1902]
**Stokes, W.** ... ... *Operative and Clinical Surgery.* (Ed. by W. Taylor) 1902
**Sultan, G.** ... ... *Atlas and Epitome of Abdominal Hernias.* (Tr. by W. B. Coley) ... ... ... ... ... ... ... ... ... 1902
**Sutton, J. Bland-** *Ligaments: their Nature and Morphology* ... 3rd Ed. 1902
**Swedenborg, E.** ... *Conjugial Love*... ... ... ... ... ... (2) New Ed. 1855
**Tilt, E. J.** ... ... *The Change of Life* ... ... ... ... ... (2) 2nd Ed. 1857
**Todd, R. B.**... ... *On Paralysis* [etc.] ... ... ... .. ... (3) 2nd Ed. 1856
**Underwood, A. S.** *Aids to Dental Anatomy and Physiology* ... 2nd Ed. 1902
**Warren and A. P. Gould, J. C.** [Eds.] *The International Text-Book of Surgery.* Vol. II. ... ... ... ... ... ... ... 1900
**Wilson, E.** ... ... *The Anatomists' Vade-Mecum* ... ... (2) 5th Ed. 1851
**Windle, B. C. A.**... *Surface Anatomy* ... ... ... ... ... ... 3rd Ed. 1902
**Yeo, I. B.** ... ... *A Manual of Medical Treatment.* 2 vols. New [3rd] Ed. [1902]

### TRANSACTIONS, REPORTS, JOURNALS, &c.

American Association of Obstetricians and Gynecologists, Transactions of the ... ... ... ... ... ... ... ... ... ... Vol. XIV. 1902
American Dermatological Association, Transactions of the, for 1901 ... 1902
American Journal of Obstetrics, The ...Vols. (1) I., II., 1869-70; XLV. 1902
American Medicine ... ... ... ... ... ... ... ... ...Vol. III. [1902]
American Public Health Association, Reports and Papers of the Vol. XXVII. 1902
Archives provinciales de Médecine ... ... ... ... ... Tome II. 1900
Archivio di Ortopedia ... ... ... ... ... ... ... ... Vol. XVIII. 1902

Boston Medical and Surgical Journal, The (1) Vols. I.-III., 1829-31; XV., 1837; XVIII., XIX., 1838-39; XXV., XXVI., 1842; XXVIII.—XXX., 1843-44; XXXII., XXXIII., 1845-46; XXXV.—LIX., 1847-59; LXII.—LXV., 1860-62; LXVII., 1863; LXIX. [1864]; LXXIV.—LXXXIX., 1866-74; XCI., 1875; XCVI.—XCVIII., 1877-78; C.—CXXV., 1879-91; CXXVIII.—CXXX., 1893-94. Vol. CXLVI. 1902

Bristol Health Report ... ... ... ... ... ... ... ... ... ... ... 1902

British Congress on Tuberculosis, London, 1901, Transactions of the, 4 vols. 1902

British Gynæcological Journal, The ... ... ... ... ... Vol. XVII. 1901

British Medical Journal, The... .. ... ... ... ... ... ... Vol. I. 1902

Buffalo Medical Journal, The... ... ... ... ... ... ... Vol. XLI. 1902

Cincinnati Lancet-Clinic, The ... ... ... ... N.S., Vol. XLVIII. 1902

Clinical Journal, The .. ... ... ... ... ... ... ... ... Vol. XIX. 1902

Clinical Society of London, Transactions of the ... ... Vol. XXXV. 1902

Columbia University, N.Y., Studies from the Department of Pathology of the College of Physicians and Surgeons... ... ... ... ... 1901-02

Edinburgh Obstetrical Society, Transactions of the... .. Vol. XXVII. 1902

Epidemiological Society of London, Transactions of the N.S., Vols. VIII.—X. 1889-91

Glasgow Medical Journal, The ... ... ... ... . ... Vol. LVII. 1902

Gynecological Society of Boston, Transactions of the (1) N.S., Vol. I. 1889

Hospital, The ... ... ... ... ... ... ... ... ... ... Vol. XXXII. 1902

HOSPITALS—

Bicêtre, L'Hospice de—Recherches sur l'Épilepsie, l'Hystérie et l'Idiotie ... ... ... ... ... ... (3) Vols. IX.—XIV. 1890-94

Royal Victoria Hospital, Montreal, Studies from the (4) Vol. I., No. iii. 1902

Journal of Medical Research, The ... ... ... Vols. VI., VII. 1901-02

Journal of the American Medical Association, The ... Vol. XXXVIII. 1902

Journal of the Sanitary Institute; with Supplement ... Vol. XXII. 1902

Lancet, The... ... ... ... ... ... .. ... ... ... ... ... ... ... Vol. I. 1902

Library World, The ... ... ... ... ... ... ... ... ... ... ... Vol. IV. 1902

Long Ashton Rural District, Annual Report for 1894 ... ... ... (3) 1895

Louisville Monthly Journal of Medicine and Surgery, The Vol. VIII. [1902]

Medical Chronicle, The ... ... .. ... ... ... ...4th Ser., Vol. II. [1902]

Medical News, The ... ... ... ... ... ... ... ... ... Vol. LXXX. 1902

Medical Press and Circular, The ... ... ... ... ... [Vol. CXXIV.] 1902

Medical Record, The ... ... ... ... ... ... ... ... ... Vol. LXI. 1902

Medical Society of the State of New York, Transactions of the ... ... 1902

Medico-Psychological Association of Great Britain and Ireland, Report of the Tuberculosis Committee of the ... ... ... ... ... ... (5) 1902

Merck's Index ... ... ... ... ... ... ... ... ... ... ... ... ... ... 1902

New York Medical Journal, The ... ... ... ... ... ... Vol. LXXV. 1902

Northumberland and Durham Medical Journal, The, for 1901 ... ... 1902

Obstetrical Transactions ... ... ... ... ... ... ... ... ... Vol. XLIII. 1902

Philadelphia Medical Journal, The ... ... ... ... ... ... Vol. IX. 1902

Practitioner, The ... ... ... ... ... ... ... ... Vol. LXVIII. 1902

| | | |
|---|---|---|
| Progressive Medicine ... ... ... ... ... ... ... ... | Vols. II., III. | 1902 |
| Royal Academy of Medicine in Ireland, Transactions of the | Vol. XX. | 1902 |
| Sanitary Record, The ... ... ... ... ... ... ... ... ... | Vol. XXIX. | 1902 |
| Scottish Medical and Surgical Journal, The ... ... ... ... | Vol. X. | 1902 |
| Virginia Medical Semi-Monthly, The ... ... ... ... (7) | Vol. VI. | 1902 |
| Year-Book of Scientific and Learned Societies ... ... ... ... ... .. ... | | 1902 |

---

We think that our readers will be glad to have the following particulars on "The Growth of the Library since 1891."

In the *Journal* for December, 1891, there appeared a full account of the beginning of our Library, and its outcome from the success of the *Journal* was clearly traced. When the Library was opened on January 5th, 1891, in a room on the premises of the Literary and Philosophic Club, at 28 Berkeley Square, several volumes and many current periodicals were available for consultation by members. The growth of the Library can be most easily seen by a tabular record compiled from the Annual Reports of the Honorary Librarian, which have been read at the meetings of the Society held at the beginning of October.

| Year. | Books. | Current Periodicals. | Duplicate volumes given to other Libraries or sold. |
|---|---|---|---|
| 1891 | 1,879 | 79 | — |
| 1892 | 2,564 | 79 | — |
| 1893 | 3,295 | 108 | — |
| 1894 | 3,776 | 108 | 1 |
| 1895 | 4,530 | 127 | 11 |
| 1896 | 5,173 | 158 | 31 |
| 1897 | 5,883 | 172 | 162 |
| 1898 | 7,180 | 170 | 63 |
| 1899 | 8,177 | 188 | 91 |
| 1900 | 8,741 | 189 | 57 |
| 1901 | 9,371 | 189 | 125 |
| 1902 | 9,898 | 189 | 68 |

On the 16th of November, 1892, the New Medical School Buildings were opened, and a committee of an equal number of representatives of the Faculty of Medicine of University College and of the Society drew up a scheme for uniting the libraries of the two institutions, for although the staff of the School had not considered that its business was to provide books either for its teachers or its students, it must in fairness be stated that they were not at that time wholly without literature, for two volumes were discovered which there is every reason to believe were the property of the School.

On the 1st of July, 1893, the Society's books were moved to the Medical School into an apartment magnificent in its proportions and decorations, but strangely inadequate for a Library, although it had been specially designed for the purpose. The walls on three sides of the room were occupied for about three-quarters of their height by windows, which, although very beautiful in themselves, considerably lessened the wall space available for bookshelves, and it was soon necessary to erect shelves against the greater part of these windows.

When the junction of the two libraries was effected a clerk was put in charge of the books, giving to their care all his time which was not engaged with School work. It was shortly afterwards found that additional help was required, and an assistant clerk was appointed. In 1894, largely through the influence of Greig Smith, nearly all the books belonging to the Royal Infirmary and the General Hospital were placed in the Library, which received a further increase by the transfer from the Museum and Library of the books which had originally belonged to the Medical Library situated in Orchard Street in the first half of the nineteenth century.

Owing to this fortunate combination, our members have access to 21,624 volumes and 227 current periodicals. These come from the following sources:—

| | Books. | | Current Periodicals. |
|---|---|---|---|
| Bristol Medico-Chirurgical Society ... | 9,898 | ... | 189 |
| Faculty of Medicine, University College | 7,829 | ... | 38 |
| Bristol Royal Infirmary ... ... ... | 2,622 | ... | — |
| Bristol General Hospital ... ... ... | 1,275 | ... | — |
| | 21,624 | ... | 227 |

Those who wish to learn, or to remind themselves of, the facts concerning the development of the Library should refer not only to our number for December, 1891, but also to that for December, 1898, in which much is told concerning its advantages.

The cataloguing is done under the card-system, having one alphabetical list of authors, subjects, and titles, with plentiful cross-references.

The label which is attached to the insides of the covers of the books to denote ownership is utterly unworthy of our excellent Library, and steps ought to be immediately taken to procure two dignified book-plates, one for each main section of the Library.

In the matter of artificial illumination, the Library is sadly behind the times; and it would be nothing less than a scandal if it is true that the installation of the electric light was frustrated only by the jealousy of one of the other departments of the School. The College authorities have done much for the Library, and they doubtless would have done more if it had not been for the crippled state of their finances.

### MEETINGS OF SOCIETIES.

### Bristol Medico-Chirurgical Society.

### Local Medical Notes.

**University College, Bristol.**—Mr. O. S. Bodman, M.B., B.S., M.R.C.S., L.R.C.P., has obtained the degree of M.D. of the Durham University; Messrs. H. Devine and W. N. Blatchford have obtained the M.R.C.S. and L.R.C.P. diplomas, and Mr. A. R. Mc Ennery the L.S.A. diploma. Messrs. A. N. Thomas and H. O. Gough were successful in the Intermediate M.B. Examination of the London University.

**The Bristol Royal Infirmary.**—The following appointments have recently been made: W. H. Harsant, F.R.C.S., Consulting Surgeon; James Swain, M.S., M.D. Lond., F.R.C.S., Honorary Surgeon; Harold F. Mole, F.R.C.S., Assistant Surgeon; C. A. Hayman, M.D. St. Andrews, F.R.C.S.I., L.D.S., Honorary Dental Assistant; John A. Nixon, B.A., M.B., B.C. Cantab., Senior Resident Officer and House Physician; E. H. Edwards Stack, M.B., B.C. Cantab., F.R.C.S., House Surgeon; H. E. Stanger-Leathes, M.R.C.S., L.R.C.P., Casualty Officer; and A. W. Conolly, M.R.C.S., L.R.C.P., Junior House Surgeon.

**The Bristol General Hospital.**—Mr. Charles Corfield has been appointed Casualty Assistant House Surgeon; Mr. C. C. C. Shaw, M.B. Lond., M.R.C.S., L.R.C.P., Assistant House Physician; Mr. J. W. Llewellyn, M.R.C.S., L.R.C.P., Assistant House Surgeon.

**The Royal Victoria Homes, Brentry.**—This useful institution forms the inebriate reformatories for various counties and boroughs, and is doing good work in our midst.

It was commenced in the year 1887, when the Rev. H. N. Burden, the present Warden of the Royal Victoria Homes, originated a scheme for the establishment of a home for the reception of criminal habitual drunkards, and a few years afterwards an institution of this sort was opened at Horfield, and in March, 1898, was licensed under the Inebriates' Act (1898) for the reception of 20 women.

In consequence of support by various county councils and municipal authorities, twenty-four of which are now subscribers, Brentry House was secured in addition, and in January, 1899, licensed for 50 female cases. The Home has rapidly increased, and is now licensed for 25 women at Horfield, which is used as the female "receiving house"; Brentry House is licensed for 200 women and 111 men; Ashford for 75 females (Roman Catholics); and in order that a better classification can be carried out, homes also in connection with Brentry have within the last few weeks been opened at Lewes and Chesterfield to receive 120 and 57 women respectively.

A few unmanageable cases can be sent by the Secretary of States' order to the State Reformatories, which have been opened at the prisons at Warwick (for men) and Aylesbury (for women).

From the Warden's Report (1901) we learn that the admissions during the year number in all 171 persons, 166 of whom were convicted under the Inebriates' Act of 1898, the remaining five persons, being two men and three women, were received under the Act of 1879. Of the persons convicted under the Act of 1898, 133 were women and 33 men. In age they range from 18 up to 70; twenty-eight of the women were under 30 years of age, forty-nine were between that age and 40, forty-one between 40 and 50, fourteen between 50 and 60, and one over 60 years of age. Three of the men were under 30, eleven between 30 and 40, ten between 40 and 50, five between 50 and 60, and four over 60 years of age.

From the report of the medical officer, Dr. H. L. Ormerod, we find that all medicines have been administered in a solid form, as by this means the tendency to frequent "sipping" is avoided, and also the desire to be on the "sick list" is diminished.

The Institution appears to be doing good work, and is under excellent management.

**Tuberculosis in Asylums**[1] (*vide* p. 368).—Phthisis is prevalent in our public asylums to an extent which calls for urgent measures.

[1] Extract from Report of the Tuberculosis Committee of the Medico-Psychological Association of Great Britain and Ireland, 1902.

A very large number of cases of phthisis have acquired that disease after admission to the asylum.

The special causes for this increased prevalence of phthisis in our asylums are in our opinion :—

Overcrowding, with consequent insufficient day, and especially night, cubic space per patient;

Insufficiency of hours in the open air ;

Defects in ventilation and heating;

Uncleanly habits ; and

Faults in dietary.

[On the other hand, it is a commonly recognised fact that in some asylums tuberculosis is so rare as to be practically unknown ; and it would appear as if admission to these asylums would be one of the best modes of treatment. It has frequently been noted how much the duration of life in the paretic patient is prolonged by the hygienic surroundings and ample feeding of a model asylum: in fact, by the best treatment of tuberculosis. —Ed.]

The means of prevention should be :—

Early diagnosis of phthisis;

Isolation of all phthisical cases ;

Limiting the size of future asylums;

Checking overcrowding;

Increasing day and night cubic space;

Restricting number of beds in dormitories;

Increased and more thorough natural ventilation per patient;

The greatest care to prevent the spread of the disease by promiscuous spitting;

A careful supervision of dietary;

Properly constructed and situated hospitals and sanatoria, with adequate and suitable surroundings for the isolation of these cases, and their treatment on the most modern lines; and

Failing such special hospitals or sanatoria, then either temporary isolation hospitals or special wards and airing courts set apart for this purpose.

### INDEX.

(R.) = Review.

Abdominal operations, A further series of intra-—Dr. J. Swain, 33.
Accommodation, Paralysis of the—W. M. Beaumont, 213.
Allchin, Dr. W. H. [Ed.]—A manual of medicine (R.), 348.
Alt, Dr. A.—Original contributions concerning the glandular structures appertaining to the human eye and its appendages (R.), 354.
American Association of Obstetricians and Gynæcologists, Transactions of the (R.), 266.
American Pediatric Society, Transactions of the (R.), 267.
American Surgical Association, Transactions of the (R.), 172.
Anæmia infantum pseudo-leukæmia, 61.
Anæmia, Splenic, 61.
Anæsthetics—Dr. F. W. Hewitt (R.), 259.
Anatomy, Surgical applied—Sir F. Treves (R.), 170.
Andrew, Dr. J. G.—Tubercular disease of the hip-joint (R.), 172.
Angina, Huchard's table, 238.
Anus, Diseases of the—D. H. Goodsall and W. E. Miles (R.), 168.
Aphorisms, definitions, reflections, and paradoxes—Dr. A. Rabagliati (R.), 254.
Appendicitis abscess, 158.
Appendicitis, Observations on cases of—T. Carwardine, 319.
Appendicitis, When to operate in—C. M. Moullin (R.), 171.
Argentine et au Paraguay, La peste bubonique dans la République (R.), 255.
Arthritis, Gonorrhœal—Dr. L. V. Jones (R.), 172.
Arthritis, Pneumococcic, 338.
Ascites due to thrombosis of the hepatic veins—Dr. T. Fisher, 209.
Assured, The causes of death among the, in the Scottish Widows' Fund and Life Assurance Society, 1874-94—Dr. C. Muirhead (R.), 267.
Asylums, Tuberculosis in, 383.
Aural practice, Golden rules of—Dr. P. R. W. de Santi (R.), 265.

Bacteriological diagnosis—Dr. W. D'E. Emery (R.), 256.
Bacteriology, Practical—Dr. H. J. Curtis (R.), 170.
Ball, Dr. J. B.—A handbook of diseases of the nose and pharynx (R.), 261.
Ballet, A. Proust and G.—The treatment of neurasthenia (R.), 361.
Bandaging, Elementary—W. Pye (R.), 267.
Bath waters—Dr. P. King (R.), 260.
Beaumont, W. M.—Paralysis of the accommodation, 213; Some cases of ptosis, 131.
Bland-Sutton; *see* Sutton.
Blood-examination as a means of diagnosis between ulcer and cancer of the stomach, 334.
Bradshaw's dictionary of bathing places (R.), 253.
Brain, The mental functions of the—Dr. B. Hollander (R.), 349.
Breast, Operation in cancer of the—C. A. Morton, 305.
Brentry, The Royal Victoria Homes, 383.
Bright's disease, Surgical treatment of chronic, 336.
Bristol: — General Hospital, 287, Fête at Clifton Zoological Gardens, 288; Smallpox in, 79; University for, 76.
Bristol Medico-Chirurgical Society, 85, 186, 282, 365, 377.
Bronchitis, Diplococcal—Dr. P. W. Williams, 135.

Burdett's hospitals and charities (R.), 257.
Burghard, W. W. Cheyne and F. F. —A manual of surgical treatment (R.), 73, 250.
Bush, J. P.—A case of gastro-jejunostomy for cancer of the pylorus, 57; The treatment of certain deformities, more especially of the nose, by the subcutaneous injection of paraffin, 220.

Cacodylates as therapeutic agents, The relative efficiency of, 243.
Calculi, The use of X-rays in the diagnosis of renal—J. Taylor, 44.
Cancer of the breast, operation in—C. A. Morton, 305.
Cancer of the stomach, 335.
Cancer of the uterus.—Dr. T. S. Cullen (R.), 352.
Carcinoma of the cervix uteri with pregnancy, Co-existence of, 160.
Carless, W. Rose and A.—A manual of surgery (R.), 360.
Carpenter, Dr. G.—Golden rules of diseases of children (R.), 253.
Carr, Dr. J. W., T. P. Pick [*et al.*]—The practitioner's guide (R.), 355.
Carter, Dr. A. H.—Elements of practical medicine (R.), 265.
Carter and Dr. F. H. Edgeworth, T. M.—On the use of morphia in uræmia, 231.
Carwardine, T.—Observations on cases of appendicitis, 319; Report on surgery, 240.
Cells, The commonwealth of—H. G. F. Spurrell (R.), 168.
Cervello, V.—The cure of pulmonary tuberculosis (R.), 265.
Chemical pathology—Dr. C. A. Herter (R.), 352.
Cheyne and F. F. Burghard, W. W. —A manual of surgical treatment (R.), 73, 250.
Children, Golden rules of diseases of—Dr. G. Carpenter (R.), 253.
Chilton, Obituary notice of Reginald Horace, 191.
Christian science—Dr. A. Moll (R.), 362.
Clarke, Dr. J. M.—Report on medicine, 333; Two cases of localised necrosis of the lung, with recovery, 122.
Climates and baths of Great Britain, The (R.), 249.
Clinical methods—Dr. R. Hutchison and H. Rainy (R.), 359.
Cocci in skin diseases, The, 245.
Columbia University, Studies from the department of pathology of the College of Physicians and Surgeons (R.), 173.
Consumption, Tendencies to—Dr. J. D. E. Mortimer (R.), 264.
Contract practice, 80.
Corns, Pathology and treatment of, 245.
Cotterell, E.—The pocket Gray (R.), 256.
Cotton, Dr. W.—Stereoscopic X-ray representation, with an example, 218.
Crile, Dr. G. W.—An experimental and clinical research into certain problems relating to surgical operations (R.), 250.
Cross, F. R.—Some landmarks in the progress of medical science (R.), 263.
Cullen, Dr. T. S.—Cancer of the uterus (R.), 352.
Curtis, Dr. H. J.—The essentials of practical bacteriology (R.), 170.

Davidson, Dr. A.—Syllabus of lectures to nurses (R.), 269.
Davies, Dr. D. S.—Notes on a case of variola nigra, 49.
Deformities, The treatment of certain, by the subcutaneous injection of paraffin.—J. P. Bush, 220.
Dermatology, An introduction to—Dr. N. Walker (R.), 361.
Dermatology, Report on—Dr. H. Waldo, 243.
Diagnosis, Physical—Gibson and Russell's (R.), 359.
Dictionary, Medical—Dr. W. A. N. Dorland (R.), 345.
Dispensing, Aids to practical—C. J. S. Thompson (R.), 361.
Dorland, Dr. W. A. N.—An American illustrated medical dictionary (R.), 345.
Dyspepsia due to adhesions, 153.

Ear, Diseases of the—T. M. Hovell (R.), 261.
Edgeworth, T. M. Carter and Dr. F. H.—On the use of morphia in uræmia, 231.
Editorial notes, 76, 176, 270, 364.
Electrolysis in the treatment of obstinate strictures of the Eustachian tube, 340.

Ellis, H.—Studies in the psychology of sex (R.), 263.
Embryology, A text-book of—Dr. J. C. Heisler (R.), 170.
Emery, Dr. W. D'E.—Handbook of bacteriological diagnosis (R.), 256.
Encyclopædia medica. — Dr. C. Watson [Ed.] (R.), 164, 345.
Endoscopy of the trachea and bronchi, 342.
Ethics, Medical—Dr. R. Saundby (R.), 357.
Ethyl bromide as a general anæsthetic, 343.
Ethyl chloride as a general anæsthetic—F. H. Rose, 53.
Eustachian tube, Electrolysis in the treatment of obstinate strictures of the, 340.
Eye, Diagnosis and treatment of the —Dr. E. Jackson (R.), 354.
Eye, The glandular structures of the human—Dr. A. Alt (R.), 354.

Fenwick, E. H.—Tumours of the urinary bladder (R.), 171.
Fernie, Dr. W. T.—Kitchen Physic (R.), 268.
"First aid," Illustrations from—Drs. Warwick and Tunstall (R.), 260.
Firth, J. L.—Report on surgery, 65.
Fischer, Dr. L.—Infant feeding in its relation to health and disease (R.), 260
Fisher, Dr. T.—A case of ascites due to thrombosis of the hepatic veins, 209; Report on medicine, 61.
Fishponds Asylum, 273.
Flemming, C. E. S.—Notes on three pathological specimens, 222.
Fox, Dr. Bonville Bradley. — In memoriam, 150.
Fox, Dr. Edward Long. — In memoriam, 97; Memorial to, 176.
Fox, R. H.—William Hunter (R.), 169.
Freyer, P. J. — Stricture of the urethra and enlargement of the prostate (R.), 253.
Frome — The New Victoria Hospital, 96.

Gastric hemorrhage, 65.
Gastric secretion, Influence of foods on, 333.
Gastritis, Pneumococcal and streptococcal, 155.
Gastro-jejunostomy, A case of—J. P. Bush, 57.
Gastrosuccorhœa, 336.
Gibson and Russell's physical diagnosis (R.), 359.
Gibson, Dr. G. A. [Ed.]—Text-book of medicine (R.), 72.
Gonorrhœal arthritis.—Dr. L. V. Jones (R.), 172.
Goodsall and W. E. Miles, D.H.—Diseases of the anus and rectum (R.), 168.
Grandin and G. W. Jarman, Drs. E. H.—A text-book of practical obstetrics (R.), 174.
Gray, The pocket — E. Cotterell (R.), 256.
Great Britain, The climates and baths of (R.), 249.
Griffiths, L. M.—The reputation of the Hotwells as a health resort, 1, 142, 193; Some things of general interest in the Bristol Medical Library, 279; On the growth of the Library of the Bristol Medico-Chirurgical Society, 376.

Händedesinfectionsfrage, Beiträge zur—Dr. R. Schaeffer (R.), 252.
Harsant, W. H.—Report on otology, 340. Resignation of, at the Bristol Royal Infirmary, 366.
Heart, Nervous affections of the, 238.
Heisler, Dr. J. C.—A text-book of embryology (R.), 170.
Hematemesis, Post-operative, 69.
Hematemesis, Vicarious, at the menstrual epoch, 69.
Hemorrhage from gastric and duodenal ulcers, 65.
Hemorrhage in cirrhosis of the liver, 68.
Hepatic veins, Ascites due to thrombosis of the—Dr. T. Fisher, 209.
Herter, Dr. C. A.—Lectures on chemical pathology (R.), 352.
Hewitt, Dr. F. W.—Anæsthetics and their administration (R.), 259.
Hiccough, Obstinate—Dr. L. F. B. Knuthsen (R.), 356.
Hip-joint, Tubercular disease of the — Dr. J. G. Andrew (R.), 172.
Hollander, Dr. B.—The mental functions of the brain (R.), 349.

Horsley, Sir Victor, at University College, Bristol, 364.
Hospitals and charities, Burdett's (R.), 257.
Hotwells (Bristol) as a health-resort, On the reputation of the—L. M. Griffiths, 1, 142, 193.
Hovell, T. M.—A treatise on the diseases of the ear (R.), 261.
Huchard's table, 238.
Hunter, William—R. H. Fox (R.), 169.
Hutchison and H. Rainy, Dr. R.—Clinical methods (R.), 359.
Hydatid disease of the lungs—Dr. A. A. Lendon (R.), 360.

Index-catalogue of the library of the Surgeon-General's Office, United States Army (R.), 264.
Infant feeding—Dr. L. Fischer (R.), 260.
Ireland, Transactions of the Royal Academy of Medicine in (R.), 258.

Jackson, Dr. E.—A manual of the diagnosis and treatment of the eye (R.), 354.
Jarman, Drs. E. H. Grandin and G. W.—A text-book of practical obstetrics (R.), 174.
Jellett, Dr. H.—A short practice of midwifery (R.), 263.
Jennings, Dr. O.—On the cure of the morphia habit without suffering (R.), 169.
Johns Hopkins hospital reports (R.), 358.
Jones, Dr. L. V.—Gonorrhœal arthritis (R.), 172.
Jones, Dr. R. L.—Vasomotor and ocular phenomena in relation to rheumatoid arthritis, 328.
Journal of obstetrics and gynæcology of the British Empire, The (R.), 175.
Jurisprudence, Medical—Dr. W. McCallin (R.), 353.

Kidney and ureter, Surgical diseases of the—H. Morris (R.), 74.
King, Dr. P.—Bath Waters (R.), 260.
Kitchen physic—Dr. W. T. Fernie (R.), 268.
Klein, Dr. E.—The diagnosis of Oriental or bubonic plague, 108.
Knopf, Dr. S. A.—Tuberculosis as a disease of the masses, and how to combat it (R.), 252.
Knuthsen, Dr. L. F. B.—Obstinate hiccough (R.), 356.

Laryngology and rhinology, Report on—Dr. P. W. Williams, 342.
Lendon, Dr A. A.—Hydatid disease of the lungs (R.), 360.
Library, Bristol Medical, Some things of general interest in the—L. M. Griffiths, 279.
Library of the Bristol Medico-Chirurgical Society, 83, 182, 277, 372; On the growth of the—L. M. Griffiths, 376.
Ligaments—J. Bland-Sutton (R.), 356.
Liver, Hemorrhage in cirrhosis of the, 68.
Local medical notes, 94, 191, 286, 382.
London, Transactions of the Obstetrical Society of (R.), 266.
Lung, Two cases of localised necrosis of the—Dr. J. M. Clarke, 122.
Lungs, Hydatid disease of the—Dr. A. A. Lendon (R.), 360.

McCallin, Dr. W.—Introduction to medical jurisprudence (R.), 353.
Makins, G. H.—Surgical experiences in South Africa, 1899-1900 (R.), 75.
Massage—Margaret D. Palmer (R.), 262; J. A. Peters (R.), 260.
Materia medica, Aids to—Dr. W. Murrell (R.), 351.
Mattison, Dr. J. B.—The Mattison method in morphinism (R.), 363.
Medical annual, The (R.), 257.
Medical life, The—G. M. Smith, 289.
Medical science, Some landmarks in the progress of—F. R. Cross (R.), 263.
Medicine, A manual of—Dr. W. H. Allchin [Ed.], 348.
Medicine, Elements of practical—Dr. A. H. Carter (R.), 265.
Medicine, Reports on—Dr. J. M. Clarke, 333; Dr. T. Fisher, 61; Dr. G. Parker, 153, 239; Dr. R. S. Smith, 235.
Medicine, Text-books of—Dr. G. A. Gibson [Ed.] (R.), 72; Dr. A. Strümpell (R.), 262.

Medico-chirurgical transactions (R.), 258.
Menstruation, Vicarious hematemesis during, 69.
Mental functions of the brain, The —Dr. B. Hollander (R.), 349.
Merck's index (R.) 362.
Merck's report (R.), 269.
Méric, H. de—Syphilis and other venereal diseases (R.), 173.
Microscope, The unreliability of the —C. H. Whiteford, 226.
Midwifery, A short practice of—Dr. H. Jellett (R.), 263.
Miles, D. H. Goodsall and W. E.—Diseases of the anus and rectum (R.), 168.
Moll, Dr. A.—Christian science, medicine, and occultism (R.), 362.
Morphia habit, On the cure of the —Dr. O. Jennings (R.), 169.
Morphia in uræmia, On the use of —T. M. Carter and Dr. F. H. Edgeworth, 231.
Morphinism, The Mattison method in—Dr. J. B. Mattison (R.), 363.
Morris, H.—Surgical diseases of the kidney and ureter (R.), 74.
Mortimer, Dr. J. D. E.—Tendencies to consumption (R.), 264.
Morton, C. A.—Report on surgery, 157; The results of operation in fifty-four cases of cancer of the breast, 305.
Moullin, C. M.—When to operate in appendicitis (R.), 171.
Moynihan, A. W. M. Robson and B. G. A.—Diseases of the stomach and their surgical treatment (R.), 74.
Muirhead, Dr. C.—Causes of death among the assured in the Scottish Widows' Fund and Life Assurance Society, 1874-94 (R.), 267.
Murrell, Dr. W.—Aids to materia medica (R.), 351.

Nasal practice, Golden rules of—Dr. P. R. W. de Santi (R.), 265.
Necrosis of the lung, Two cases of localised—Dr. J. M. Clarke, 122.
Nephropexy, 241.
Nephroptosis, The indications in—Dr. J. Swain, 315.
Neurasthenia, The treatment of—A. Proust and G. Ballet (R.), 361.
New York, Transactions of the Medical Society of the State of (R.), 357.
Nose, The accessory sinuses of the —Dr. A. L. Turner (R.), 168.
Nose, Diseases of the—Dr. J. B. Ball (R.), 261.
Nose, Measurements for operating distances in the, 344.
Nose, Treatment of deformities of the, by subcutaneous injection of paraffin—J. P. Bush, 220.
Nurses, Syllabus of lectures to—Dr. A. Davidson (R.), 269.
Nursing experiences on the "hospital ships" at Dartford, 177.

Obituary, 97, 105, 191.
Obstetrical Society of London, Transactions of the (R.), 266.
Obstetrics, Practical—Drs. E. H. Grandin and G. W. Jarman (R.), 174.
Obstetrics, Report on—Dr. W. C. Swayne, 160.
Œsophagus, Ulcer of the, 155.
Ophthalmic optics, Elementary—J. H. Parsons (R.), 354.
Otology, Report on—W. H. Harsant, 340.

Paget, Sir James—Memoirs and letters of (R.), 248; Selected essays and addresses by (R.), 249.
Palmer, Margaret D.—Lessons on massage (R.), 262.
Pancreas, Peripheral reflex secretion of the, 239.
Paraffin, Subcutaneous injection of, in the treatment of certain deformities, more especially the nose —J. P. Bush, 220.
Paralysis of the accommodation—W. M. Beaumont, 213.
Parker, Dr. G.—Reports on medicine, 153, 239.
Parsons, J. H.—Elementary ophthalmic optics, (R.), 354.
Pasteur, The life of—R. Vallery-Radot (R.), 70.
Pathological specimens, Notes on three—C. E. S. Flemming, 222.
Peritoneum, Tuberculosis of the, 157.
Peters, J. A.—Manipulation (or massage) (R.), 260.
Pharmacopœia of the hospital for diseases of the throat, nose, and ear (R.), 362.
Philadelphia, Transactions of the College of Physicians of (R.), 258

Pick, T. P., Dr. J. W. Carr [*et al.*]—The practitioner's guide (R.), 355.
Plague: A treatise on—Major G. S. and Dr. J. Thomson (R.), 255; La peste bubonique dans la République Argentine et au Paraguay (R.), 255.
Plague, The diagnosis of Oriental or bubonic—Dr. E. Klein, 108; Editorial note on, 177.
Pneumococcic arthritis, 338.
Poland, J.—A retrospect of surgery during the past century (R.), 265.
Practitioner's guide, The—Dr. J.W. Carr, T. P. Pick [*et al.*], 355.
Pregnancy, Co-existence of carcinoma of the cervix uteri with, 160.
Pregnancy, Primary ovarian, 240.
Proust and G. Ballet, A.—The treatment of neurasthenia (R.), 361.
Pseudo-leukæmia, Anæmia infantum, 61.
Psychology of sex—H. Ellis (R.), 263.
Ptosis, Some cases of—W. M. Beaumont, 131.
Pye, W.—Elementary bandaging and surgical dressing (R.), 267.

Rabagliati, Dr. A.—Aphorisms, definitions, reflections, and paradoxes (R.), 254.
Rainy, Dr. R. Hutchison and H.—Clinical methods (R.), 359.
Rayner, D. C.—Tubal abortion, 323.
Reichmann's disease, 336.
Rheumatoid arthritis, Vasomotor and ocular phenomena in relation to—Dr. R. L. Jones, 328.
Rhinology, Report on laryngology and—Dr. P. W. Williams, 342.
Robson and B. G. A. Moynihan, A. W. M.—Diseases of the stomach and their surgical treatment (R.), 74.
Roentgen ray: X-rays in the diagnosis of renal calculi—J. Taylor, 44; Stereoscopic X-ray representation—Dr. W. Cotton, 218.
Rose, F. H.—Notes on ethyl chloride as a general anæsthetic, 53.
Rose and A. Carless, W.—A manual of surgery (R.), 360.
Ruata, Dr. C.—Pulmonary tuberculosis (R.), 265.
Russell's physical diagnosis, Gibson and (R.), 359.
Sanatorium treatment of consumption, 81.
Sanitary inspector's handbook, The—A. Taylor (R.), 269.
Santi, Dr. P. R. W. de—Golden rules of aural and nasal practice (R.), 265.
Saundby, Dr. R.—Medical ethics (R.), 357.
Schaeffer, Dr. R.—Experimentelle und kritische beiträge zur händedesinfectionsfrage (R.), 252.
Senn, N.—Principles of surgery (R.), 170.
Sex, Psychology of—H. Ellis (R.), 263.
Sick, Preparations for the, 82, 179, 275, 368.
Smallpox in Bristol, 79.
Smith, G. M.—The medical life, 289.
Smith, Dr. R. S.—Report on medicine, 235.
Smithsonian report (R.), 72, 258.
Society, Bristol Medico-Chirurgical, 85, 186, 282, 365, 377.
South Africa, Surgical experiences in, 1899–1900—G. H. Makins (R.), 75.
Spleen, Acute tuberculosis of the, 64.
Splenic anæmia, 61.
Spurrell, H. G. F.—The commonwealth of cells (R.), 168.
Staphylococcus vaccine, Inoculation of, for furunculosis, sycosis, acne, 247.
Stereoscopic X-ray representation—Dr. W. Cotton, 218.
Stomach, Acute dilatation of the, 156; Dr. H. C. Thomson (R.), 356.
Stomach, Blood-examination as a means of diagnosis between ulcer and cancer of the, 334.
Stomach, Cancer of the, 335.
Stomach, Diseases of the, 153; A. W. M. Robson and B. G. A. Moynihan (R.), 74.
Stomach, Fat-splitting ferment in, 333.
Stomach hour-glass, 334.
Stomach, Syphilitic affections of the, 154.
Stomach, Tubercular ulcers of the, 239.
Stomach, Ulcer of the, 334.
Stricture of the urethra—P. J. Freyer (R.), 253.
Strümpell, Dr. A.—A text-book of medicine (R.), 262.
Surgery, A manual of—W. Rose and A. Carless (R.), 360.

Surgery during the past century, A retrospect of—J. Poland (R.), 265.
Surgery, Principles of—N. Senn (R.), 170.
Surgery, Reports on—T. Carwardine, 240; J. L. Firth, 65; C. A. Morton, 157; Dr. J. Swain, 336.
Surgical operations, On certain problems relating to—Dr. G. W. Crile (R.), 250.
Surgical treatment—W. W. Cheyne and F. F. Burghard (R.), 73, 250.
Sutton, J. Bland-—Ligaments (R.), 356; Tumours, innocent and malignant (R.), 171.
Swain, Dr. J.—A retrospect of a further series of intra-abdominal operations, 33; Report on surgery, 336; The indications for treatment in nephroptosis, 315.
Swayne, Dr. W. C.—Report on obstetrics, 160.
Symes, Dr. J. O.—On some urinary affections, with especial reference to their treatment by urotropin, 27.
Syphilis—H. de Méric (R.), 173.
Syphilitic affections of the stomach, 154.

Taylor, A.—The sanitary inspector's handbook (R.), 269.
Taylor, J.—The use of X-rays in the diagnosis of renal calculi, 44.
Tendon grafting, 159.
Thompson, C. J. S.—Aids to practical dispensing (R.), 361.
Thomson, Major G. S. and Dr. J. —A treatise on plague (R.), 255.
Thomson, Dr. H. C.—Acute dilatation of the stomach (R.), 356.
Thrombosis, 235.
Thrombosis of the hepatic veins, A case of ascites due to—Dr. T. Fisher, 209.
Treatment, Medical—Dr. I. B. Yeo (R.), 358.
Treves, Sir F.—Surgical applied anatomy (R.), 170.
Tubal abortion.—D. C. Rayner, 323.
Tuberculosis—V. Cervello (R.), 265; Dr. S. A. Knopf (R.), 252; Dr. C. Ruata (R.), 265.
Tuberculosis, Crusade against, 176.
Tuberculosis in asylums, 383.
Tumours—J. Bland-Sutton (R.), 171.
Tumours of the urinary bladder—E. H. Fenwick (R.), 171.
Tunstall, Drs. Warwick and—Illustrations from "first aid" to the injured and sick (R.), 260.
Turner, Dr. A. L.—The accessory sinuses of the nose (R.), 168.

Ulcers of the stomach, 334; Tubercular, 239.
University of Bristol, A, 76.
Uræmia, On the use of morphia in —T. M. Carter and Dr. F. H. Edgeworth, 231.
Urethra, Stricture of the—P. J. Freyer (R.), 253.
Urinary affections, Urotropin in the treatment of some—Dr. J. O. Symes, 27.
Urinary bladder, Tumours of the—E. H. Fenwick (R.), 171.
Urotropin in the treatment of some urinary affections—Dr. J. O. Symes, 27.
Uterus, Cancer of the—Dr. T. S. Cullen (R.), 352.

Vallery-Radot, R.—The life of Pasteur (R.), 70.
Variola nigra, Notes on a case of—Dr. D. S. Davies, 49.

Waldo, Dr. H.—Report on dermatology, 243.
Walker, Dr. N.—An introduction to dermatology (R.), 361.
Warwick and Tunstall, Drs.—Illustrations from "first aid" to the injured and sick (R.), 260.
Watson, Dr. C. [Ed.]—Encyclopædia medica (R.), 164, 345.
Whiteford, C. H.—The unreliability of the microscope in the diagnosis of malignant disease, 226.
Williams, Dr. P. W.—Diplococcal bronchitis, 135; Report on laryngology and rhinology, 342.
Winsley, Proposed sanatorium at, 367.

X-rays—*See* Roentgen ray.

Yeo, Dr. I. B.—A manual of medical treatment (R.), 358.

J. W. ARROWSMITH, Printer, Quay Street, Bristol.

